# Increased *ATP2A1* Predicts Poor Prognosis in Patients With Colorectal Carcinoma

**DOI:** 10.3389/fgene.2022.661348

**Published:** 2022-06-16

**Authors:** Guoshun Zhang, Hua Shang, Bin Liu, Guikai Wu, Diyang Wu, Liuqing Wang, Shengnan Li, Zhiyuan Wang, Suying Wang, Juxiang Yuan

**Affiliations:** ^1^ School of Public Health, North China University of Science and Technology, Tangshan, China; ^2^ Department of Gastroenterology, Affiliated Hospital of North China University of Technology, Tangshan, China; ^3^ Blood Purification Department of Tangshan Infectious Disease Hospital, Tangshan, China; ^4^ Department of Gastroenterology, Chaisang District People’s Hospital, Jiujiang, China; ^5^ Department of Gastroenterology, Tangshan Workers’ Hospital, Tangshan, China; ^6^ Department of Gastroenterology, Hongci Hospital, Tangshan, China

**Keywords:** ATP2A1, CRCs, prognosis, oncogene, immune infiltration

## Abstract

Colorectal cancer is one of the most common malignant tumors in the digestive system. Traditional diagnosis and treatment methods have not significantly improved the overall survival of patients. In this study, we explored the value of *ATP2A1* as a biomarker in predicting the prognosis of colorectal cancer patients. We used the TCGA database to reveal the relationship between *ATP2A1* mRNA level and prognosis, methylation, and immune invasion in colorectal cancer. The results showed that the expression of *ATP2A1* was increased in colorectal cancer. The overall survival of patients with high expression of *ATP2A1* was significantly lower than patients with low expression of *ATP2A1*. Cox regression analysis showed that high expression of *ATP2A1* was an independent risk factor for poor prognosis in colorectal cancer patients. In addition, we used three datasets to perform a meta-analysis, which further confirmed the reliability of the results. Furthermore, we revealed that *ATP2A1* could significantly inhibit the proliferation of colorectal cancer cells by inhibiting the autophagy process and was associated with several immune cells, especially CD8 + T cells. Finally, four small molecule drugs with potential inhibition of *ATP2A1* expression were found by CMap analysis. This study demonstrates for the first time that *ATP2A1* is a potential pathogenic factor, which may play a significant role in colorectal cancer.

## Introduction

Colorectal cancer is the third most common cancer and the fourth most frequent cause of cancer deaths globally. More than 1 million patients have colorectal cancer every year (Weitz et al., 2005; Cunningham et al., 2010). Early screening of colorectal cancer (CRC) has made considerable progress in many countries. In addition, many factors, such as drugs, diet, and exercise, have largely prevented the occurrence of this disease (Ferlay et al., 2015). Current treatments include open and laparoscopic surgery, radiotherapy, neoadjuvant, and palliative chemotherapy. However, progress has been slow on the therapeutic front, and the overall survival of patients with colorectal cancer has not improved significantly (Brody, 2015). Hence, researches are urgently needed to discover new pathological factors that can be used to unravel the complex pathological mechanisms of colorectal cancer in the hope of improving the prognosis of CRC patients.

Intracellular calcium is an important part of cell physiological activities. The dynamic calcium changes contribute to cell growth and intercellular communication (Azeez et al., 2018). Abnormal Ca^2+^ homeostasis is associated with a series of diseases, such as diabetes, cardiovascular disease, and chemoresistance of cancer (Kucukkaya et al., 2019). A series of enzymes mainly regulate intracellular Ca^2+^ concentrations. Principally, the sarcoplasmic reticulum Ca^2+^ ATPases (SERCA) family actively transports Ca^2+^ from the cytosol to the SER. SERCA contains 14 isoforms and controls cell death and survival (Chemaly et al., 2018). *SERCA1* is an isoform of *ATP2A1*, short for Endoplasmic Reticulum Ca^2+^ Transporting which contributes to calcium sequestration involved in muscular excitation/contraction (Odermatt et al., 2000). However, more physiological and pathological functions of *ATP2A1* are unknown, especially in colorectal cancer.

It is of great significance to identify important regulatory molecules in the pathological process and develop targeted drugs that can delay the malignant progression of the tumor (Santos et al., 2017). For example, the overexpression of the epithelial growth factor receptor (EGFR) can promote the neovascularization of tumor tissue and promote tumor proliferation and migration; however, anti-EGFR monoclonal antibodies have been used clinically for several years to kill tumor cells and delay the malignant progression of tumor patients (Santos et al., 2021). Furthermore, the connectivity map (CMap) database can closely link the expression profile of abnormal genes of diseases with potential corrective drugs (Lamb et al., 2006). For example, several drugs inhibiting the proliferation of tumor cells were found in the connectivity map database by using differentially expressed genes in medulloblastoma (Liu et al., 2020). Therefore, targeted drugs to correct the disorder of gene expression can be quickly found through the CMap database, and this exploration can be used as an engine to guide more researchers to focus on tumor drug research.

In this study, we attempted to reveal the relationship between *ATP2A1* expression and prognosis in colorectal cancer patients by bioinformatics analysis. First, we analyzed the difference of the *ATP2A1* mRNA expression in 42 paracancerous tissues and 488 cancer samples using TCGA data and prepared a K-M survival curve; univariate and multivariate analysis was used to evaluate the relationship between *ATP2A1* expression and prognosis. Then, we used the TCGA data to evaluate the relationship between *ATP2A1* methylation level and gene expression and clinical features. To generate reliable results, we also conducted a meta-analysis using three data sets to evaluate the overall prognostic value of *ATP2A1*. In addition, the correlation between *ATP2A1* expression and immune infiltration was evaluated using the Tumor IMmune Estimation Resource (TIMER) database. Subsequently, several small molecule drugs that can inhibit the expression of *ATP2A1* were found in the CMap database, which may become a potential drug for the treatment of colorectal cancer. Finally, we confirmed the effect of *ATP2A1* on the biological behavior of colorectal cells *in vitro*. Therefore, this study uses adequate evidence to demonstrate the value of *ATP2A1* in the prognosis of colorectal cancer and provides a new perspective for the pathogenesis and drug treatment of colorectal cancer.

## Materials and Methods

### Data Collection

The Cancer Genome Atlas (TCGA) database (https://portal.gdc.cancer.gov/) is a public database dedicated to oncology research, containing the original data of 33 human tumors such as transcriptome profiling, copy number variation, and DNA methylation data. In addition, the database allows researchers to download various types of tumor data for re-analysis to promote oncology research (Liu et al., 2021). This study downloaded DNA methylation data, RNA sequencing data, and corresponding clinical information of patients with colorectal cancer from the TCGA database (workflow type: HTSeq-FPKM). The data included 42 paracancerous samples and 488 tumor samples of colorectal cancer, and patients lacking key clinical information were removed. Finally, 339 CRC samples with complete clinical information were retained to explore the prediction of *ATP2A1* on the survival, prognosis, and biological function of patients with colorectal cancer (Supplementary Table S1). Moreover, we collected two datasets from the GEO database, GSE41258 (186 colon cancer tissues and 54 normal tissues) and GSE39582 (566 colon cancer tissues and 19 normal tissues), to analyze the expression of *ATP2A1,* GSE39582 was further used to explore the relationship between *ATP2A1* expression and pathological features of CRC.

### The GO and KEGG Functional Analysis of *ATP2A1*

The Gene Oncology (GO) and Kyoto Encyclopedia of Genes and Genomes (KEGG) databases are the two most authoritative databases for gene function research and are the two most commonly used enrichment analysis methods (Liu et al., 2020). In oncology research, the above two analysis methods can predict the biological function of target genes in the course of disease according to the overall expression level of transcriptomics. Therefore, this study attempts to reveal the regulatory effect of *ATP2A1* in the colorectal pathological process through these two analytical methods. First, according to the median expression level of *ATP2A1* in RNA sequencing data of colorectal patients, the patients were artificially divided into high and low expression groups of *ATP2A1*. Second, the Wilcox test was used to obtain the differential expression data between the two groups with logFC ≥1 and *p* value less than 0.05 using R software. Finally, the genes with differences between the two data groups were used for the functional enrichment analysis of GO and KEGG and the images through the “clusterProfiler” program of R software (Tan et al., 2020). The results were shown in a bubble chart, the color represented q-value, and the size of the dot represented the count number.

### TIMER Database Analysis

The tumor immune microenvironment has an determinative regulatory effect on the prognosis of patients. Moreover, previous studies have shown that the expression disorder of pathogenic genes can affect the rate of immune cell infiltration in the tumor microenvironment (Tan et al., 2020). The Tumor IMmune Estimation Resource (TIMER) is a visual web tool for studying multiple cancer types and immune interactions and provides six main analysis modules to interactively explore the relationship between immune infiltration and multiple factors (https://cistrome.shinyapps.io/timer/) (Li et al., 2017). First, this study explored the relationship between the mRNA expression level of *ATP2A1* and the expression of six different tumor-infiltrating immune cells in the gene module of the TIMER database. Then, we explored the effect of *ATP2A1* expression level on the somatic copy number alterations of infiltrating immune cells in the SCNA module of the TIMER database. Finally, in the correlation module of the TIMER database, we explored the expression relationship between *ATP2A1* mRNA expression level and the immune checkpoints (PD-1 and PD-L1). The images of analysis results made in the TIMER database were provided on the official website of the TIMER database, and *p* < 0.05 was considered statistically significant.

### Meta-Analysis

PubMed, Web of Science, and Embase databases did not produce articles about *ATP2A1* and the prognosis of colorectal cancer patients. Therefore, we could not obtain the relationship between *ATP2A1* and the prognosis of colorectal cancer patients to be included in the meta-analysis. To confirm the impact of high expression of *ATP2A1* on the prognosis of colorectal patients, we used data stored in the public database to complete a meta-analysis to confirm the risk of *ATP2A1* on the prognosis of colorectal patients. Therefore, we selected three groups of datasets with survival times of CRC patients from the TCGA (339 patients) and GEO databases [GSE39582 (566 patients); GSE71187 (52 patients)] to perform the meta-analysis. The above three datasets used the Cox regression model individually to obtain the hazard ratio value for the prognosis of patients with colorectal cancer. We pooled the data with *ATP2A1* expression from the above databases, and efficacy was revealed as a hazard ratio (HR) with a 95% confidence interval (CI). Q test (I^2^ statistics) was used to assess the heterogeneity between the three datasets. A random-effects model would be selected if I^2^ > 50%; otherwise, a fixed-effects model was applied. The meta-analysis was completed using the R software (Tan et al., 2020).

### CMap and PubChem

The connectivity map (CMap) is a series of genomic transcription and expression data derived from cultured human cells processed by bioactive small molecules and pattern matching algorithms (https://portals.broadinstitute.org/CMap/). These algorithms can find the functional relationship between drugs, genes, and diseases (Lamb et al., 2006; Lamb, 2007). The database contains information about 1,309 compounds. In this study, the Pearson correlation coefficient was used to screen the positive and negative genes co-expressed with *ATP2A1*, regarded as up-regulated and down-regulated genes, respectively. Then, they were uploaded to the CMap database to analyze the drugs negatively related to the target genes, which may be used as candidate drugs for the inhibition of *ATP2A1* expression (*p* < 0.05, enrichment < −0.80). The 2D and 3D structures and molecular formulas of the small-molecule drugs were obtained from the PubChem database (https://pubchem.ncbi.nlm.nih.gov/).

### Cell Treatment

Cell lines such as human normal colorectal mucosal cells FHC and colorectal cancer cells (HCT116 and SW480) were obtained from the American Type Culture Collection. All cells were cultured in a complete medium consisting of 89% RPMI 1640 medium, 10% fetal bovine serum, and 1% Penicillin/Streptomycin. Cells were cultured at a constant temperature of 37°C and 5% carbon dioxide for further treatment. Selected SW480 cells were treated with a short-hairpin RNA (shRNA) for silencing *ATP2A1*. Negative control shRNA (shNC, 5'- UUC​UCC​GAA​CGU​GUC​ACG​UTT-3') and shRNA targeting *ATP2A1* (shATP2A1, 5'-AAA​AUA​GGC​CAA​ACA​UUC​CUC-3') were transfected into the SW480 cells and cultured for 48h, and then the cells were used for further experiments.

### CCK8 and Colony Formation

For verifying whether the proliferation ability of *ATP2A1* knockdown cells is affected, shRNA-treated cells were planted in 96 well plates with 2000 cells per well and then cultured in a complete medium. An appropriate amount of cell counting kit-8 (CCK8) reagent was added at the selected time points, and the culture was continued for 4 h. Finally, the absorbance value of cells was detected by a multifunctional microplate reader (BioTek, US) at 460 nm.

In addition, we further verified the proliferation ability of transfected cells by a colony formation experiment. We planted 10^3^ cells in a 6-well plate and then cultured them in a complete medium for 14 days. Finally, colony formations were fixed and stained with crystal violet solution. All experiments were independently repeated three times. The data were statistically analyzed and mapped by Prism 9.

### Real-Time PCR and Western Blotting

The RNA of all cells was extracted according to the protocol of the RNA extraction kit (Invitrogen, United States) and then transformed into cDNA by reverse transcription reaction. Real-time PCR (RT-PCR) was then performed using the same amount of cDNA samples, and the expression of *ATP2A1* was detected by specific designed primers *ATP2A1*-F (5'-CCG​GAC​CAA​GTT​AAG​CGG​AA-3') and *ATP2A1*-R (5'-TCA​CCT​TCC​TCA​AAC​CAG​GC-3'), as well as GAPDH-F (5'-CAA​GGT​CAT​CCA​TGA​CAA​CTT​TG-3') and GAPDH-R (5'-GTC​CAC​CAC​CCT​GTT​GCT​GTA​G-3'). After incubation with RIPA lysis buffer, all proteins were obtained from cells, then loaded and analyzed by SDS-PAGE. Next, the protein samples were transferred to the PVDF membrane and sealed with 5% skimmed milk. In addition, the PVDF membrane was incubated with diluted specific primary antibodies according to the protocol, and the corresponding secondary antibodies conjugated with HRP were performed after PBST cleaning. Finally, protein bands were displayed by luminescent solution and recorded by the ECL system.

### Statistical Analysis

Statistical analysis was performed with R (4.0.3). The dataset was divided into low expression and high expression groups according to the median expression level of *ATP2A1* mRNA. The Kaplan-Meier analysis was used to evaluate the relationship between *ATP2A1* expression and prognosis. Univariate and multivariate Cox regression models were used to predict whether *ATP2A1* was an independent prognostic risk factor. Pearson correlation coefficient was used to screen the co-expression genes of APT2A1. *p* < 0.05 was considered statistically significant.

## Results

### High Expression of *ATP2A1* Correlates With Clinical Features and Poor Prognosis in Colorectal Cancer

**FIGURE 1:**
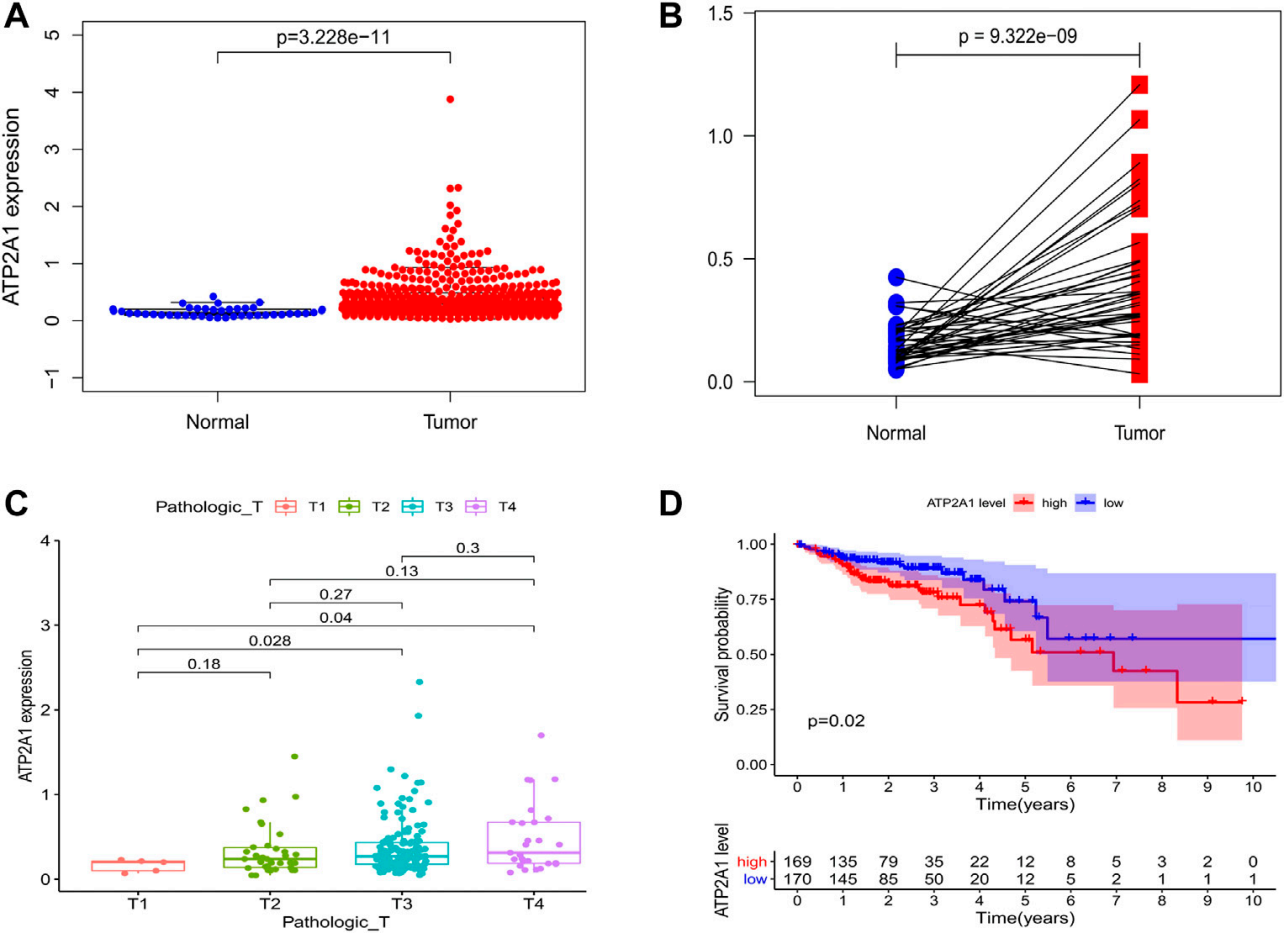
Bioinformatics analysis of *ATP2A1* expression and survival in colorectal cancer patients. **(A)** The expression of *ATP2A1* in paracancerous tissues was lower than that in tumor tissues in TCGA dataset (*p* = 3.228e-11). **(B)** Paired analysis of *ATP2A1* expression of colorectal patients in TCGA database (*p* = 9.322e-09). **(C)** Correlations of *ATP2A1* expression with Pathologic T (T3 vs. T1, *p* = 0.028; T4 vs. T1, *p* = 0.04). **(D)** KM survival analysis of *ATP2A1* high expression group and low expression group (*p* = 0.02).

Firstly, we explored the mRNA expression level of *ATP2A1* in paracancerous and tumor groups of colorectal cancer in the TCGA dataset, as well as in normal tissues and colon cancer tissues in the GEO dataset. We found that the expression level of *ATP2A1* was significantly increased in colorectal cancer patients (Figure 1A) and colon cancer patients (Supplementary Figures S1A,B). However, there may be statistical deviation due to the imbalance between the tumor tissue samples and paracancerous tissues. Subsequently, paired analysis was performed, and we found that the expression level of *ATP2A1* in tumor samples of the same patient was significantly higher than that in the paracancerous samples (Figure 1B). Further, the chi-squared test was used to explore the relationship between the mRNA expression level of *ATP2A1* and clinical features of colorectal cancer patients, and the results showed that *ATP2A1* expression was significantly higher in T3 and T4 tumors than in T1 (*p* < 0.05) in TCGA database (Figure 1C), and *ATP2A1* expression was higher in stage 2 and stage 3 tumor than stage 1 (Supplementary Figure S1C), and higher in T2, T3 and T4 tumors than in T1 (*p* < 0.05) in GEO database (Supplementary Figure S1D). Consequently, we speculated that *ATP2A1* might be involved in the progression of colorectal cancer. Therefore, the final Kaplan Meier survival analysis showed that the prognosis of the *ATP2A1* high expression group was significantly worse than the *ATP2A1* low expression group (Figure 1D). Thus, the above results show that the abnormally high expression of *ATP2A1* in colorectal cancer can lead to poor prognosis, which may play an important regulatory role in the malignant process of colorectal cancer.

### Elevated *ATP2A1* is an Independent Predictor of Poor Prognosis in Patients With Colorectal Cancer

**FIGURE 2:**
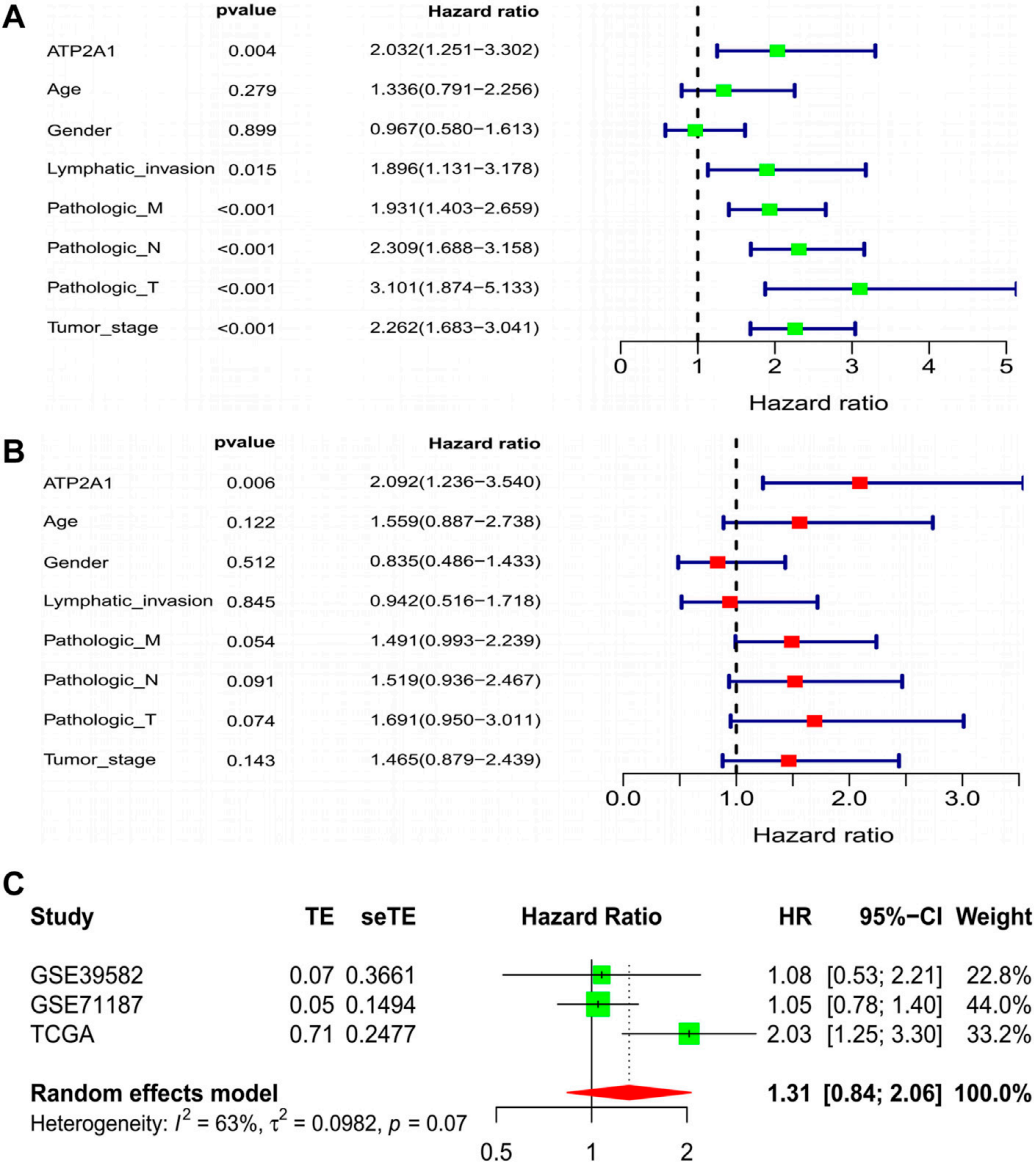
Cox regression model and meta-analysis of *ATP2A1* expression in CRCs. **(A)** Univariate analysis of *ATP2A1* expression and clinical related factors [*ATP2A1*, *p* = 0.004, HR = 2.032 (95%CI (1.251-3.302))]. **(B)** Multivariated analysis of *ATP2A1* expression and clinical related factors [*ATP2A1*, *p* = 0.006, HR = 2.092 (95%CI (1.236-3.540))]. **(C)** Forest plots of meta-analysis of *ATP2A1* effect on prognosis from multiple data sets [HR = 1.31 (95%CI (0.84-2.06))].

In order to explore whether the clinical characteristics of patients interfere with the impact of *ATP2A1* on the prognosis of patients, the Cox model was used to comprehensively consider if *ATP2A1* could be used as an independent risk factor for patients with colorectal cancer. Univariate analysis showed that high expression of *ATP2A1* resulted in poor prognosis in patients with colorectal cancer [*p* < 0.01, hazard ratio (HR) = 2.032 (95%CI (1.251–3.302))] and TNM stage, lymphatic invasion, and tumor stage are also independent risk factors [*p* < 0.01, hazard ratio (HR > 1)] (Figure 2A). We then conducted a multivariate analysis that showed that *ATP2A1* was also an independent risk factor of poor prognosis [*p* < 0.01, hazard ratio (HR) = 2.092 (95%CI (1.236–3.540))] (Figure 2B). Therefore, the adverse effect of *ATP2A1* on the prognosis of colorectal patients is an inevitable factor, not accidental. However, to avoid the deviation caused by the single data analysis results, we combined the three databases to complete a meta-analysis verifying the impact of *ATP2A1* on the prognosis of patients with colorectal cancer. The meta-analysis results show that the single HR value of the three databases is greater than 1, and the pooled HR and 95% CI was 1.31 (0.84–2.06) (Figure 2C). Thus, the risk of poor prognosis of patients with colorectal cancer with *ATP2A1* was determined.

### Regulatory Effect of Methylation Sites on *ATP2A1* Expression

**FIGURE 3:**
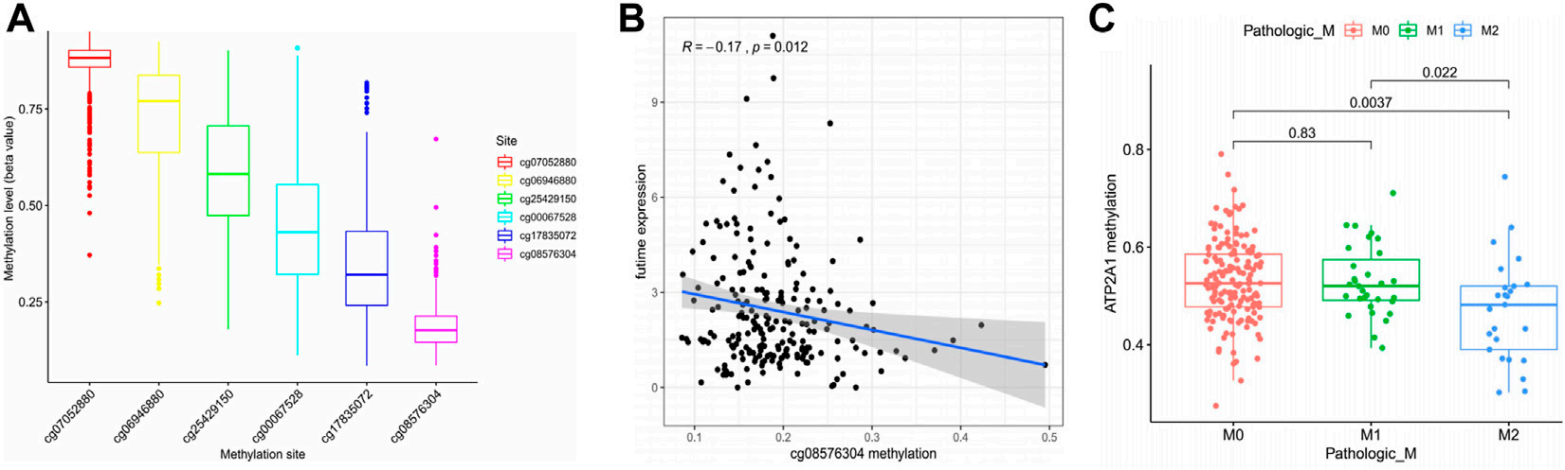
Regulation of methylation level on *ATP2A1* expression. **(A)** The methylation level of six *ATP2A1* DNA promoter CpG sites. **(B)** The expression of *ATP2A1* is negtively related with the methylation of cg08576304. **(C)** Correlationship between *ATP2A1* methylation level and pathologic M (M2 vs. M0, *p* = 0.0037; M2 vs. M1, *p* = 0.022).

As one of the important regulatory forms of epigenetics, methylation regulation significantly impacts gene expression. Therefore, this study attempts to demonstrate whether the high expression of *ATP2A1* in patients with colorectal cancer is regulated by its methylation sites. Firstly, the DNA methylation data of colorectal patients were downloaded from the TCGA database, and six different methylation sites were found to regulate the expression of *ATP2A1* (Figure 3A). Further, co-expression analysis showed that *ATP2A1* was negatively regulated only by cg08576304 among the six different methylation sites (Figure 3B). In addition, the analysis of clinically related properties found that the decrease of the methylation status of the methylation site of *ATP2A1* could promote tumor metastasis (Figure 3C). The methylation status based on cg08576304 is the lowest among the six different methylation sites and has a significant negative correlation with the mRNA level of *ATP2A1*. Therefore, the low methylation status of cg08576304 promotes the high expression of *ATP2A1* in the tissues of patients with colorectal cancer.

### Relationships of *ATP2A1* With Immune Infiltrations and PD1

**FIGURE 4:**
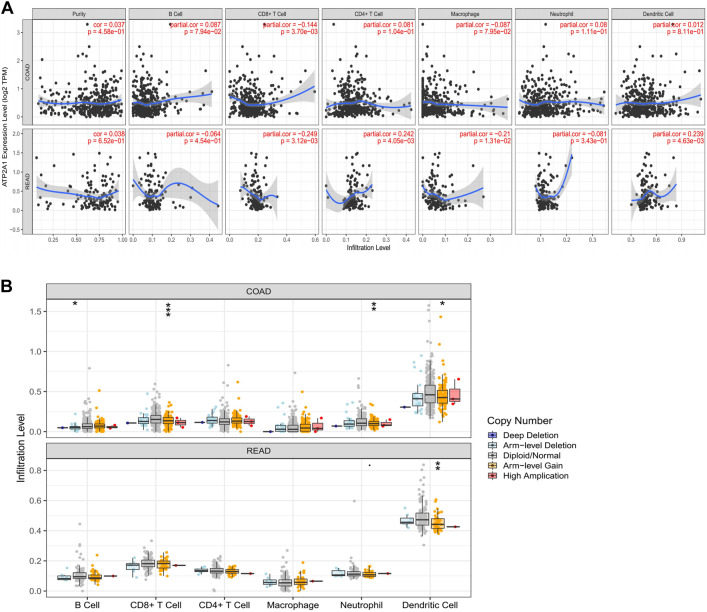
The expression of *ATP2A1* was related to immune infiltration. **(A)** The relationship between *ATP2A1* mRNA level and the the infiltration of six immune cells in COAD and READ. **(B)** The relationship between copy number variation of *ATP2A1* and multiple immune cells. *p < 0.05, **p < 0.01, ***p < 0.001.

We used the TIMER database and GSE39582 dataset to evaluate the correlations of *ATP2A1* expression and immune infiltrations. As illustrated in Figure 4A, the expression of *ATP2A1* was negatively correlated with immune infiltration of CD8^+^ T cells whether in colon adenocarcinoma (COAD) (*r* = −0.144, *p* = 3.70e–03) or in rectum adenocarcinoma (READ) (*r* = −0.249, *p* = 3.12e–03), and positively correlated with CD4^+^ T cells (*r* = 0.242, *p* = 4.05e–03), dendritic cells (*r* = 0.239, *p* = 4.63e–03) in READ, and negatively correlated with macrophage (*r* = −0.21, *p* = 1.31e–02). The same trend is verified in the GSE39582 as follows: negative correlation between *ATP2A1* expression with macrophage (*PTGS2*, *r* = −0.19, *p* = 3.3e–06; *CD163*, *r* = −0.19, *p* = 5.7e–06; *MS4A4A*, *r* = −0.23, *p* = 1.78e–08; *VSIG4*, *r* = −0.1, *p* = 1.6e–02) (Supplementary Figures S3E–H), and positive correlation with B cell (*CD19*, *r* = 0.12, *p* = 4.9e–03; *CD79A*, *r* = 0.094, *p* = 2.5e–02) (Supplementary Figures S3A,B)and dendritic cell (*ITGAX*, *r* = 0.093, *p* = 2.7e–02) (Supplementary Figure S3K). Then, we explored the copy number changes of *ATP2A1* in different immune cells. Arm-level gain was the most common form in colorectal cancer (*p* < 0.05) (Figure 4B). Collectively, the above results showed that high expression of *ATP2A1* might affect the prognosis of colorectal cancer partly through immunity. Moreover, due to the great prospect of immunotherapy, we also examined the relationship between *ATP2A1* and common immune checkpoints. A positive correlation of *ATP2A1* with PD1 (enconded by *PDCD1*) (Supplementary Figures S2A,B) in COAD (*r* = 0.174, *p* = 4.22e–04) and READ (*r* = 0.313, *p* = 1.78e–04) was observed. We also noticed the same positive relationship between *ATP2A1* and PD-L1 (enconded by *CD274*) (Supplementary Figures S2C,D) in COAD (*r* = 0.103, *p* = 3.76e–02) and READ (*r* = 0.195, *p* = 2.12e–02). Meanwhile, the positive correlations between *ATP2A1* with *PDCD1* (*r* = 0.24, *p* = 9.2e–09) and *CD274* (*r* = 0.3, *p* = 9.6e–13) were also proved based on GSE39582 dataset (Supplementary Figures S3L,M). There is a positive co-expression relationship between *ATP2A1* and PD-L1 and PD1. Whether *ATP2A1* can become a biological target of immunotherapy needs to be confirmed by more studies.

### Functional Annotations and Signaling Pathway Enrichment of *ATP2A1*

**FIGURE 5:**
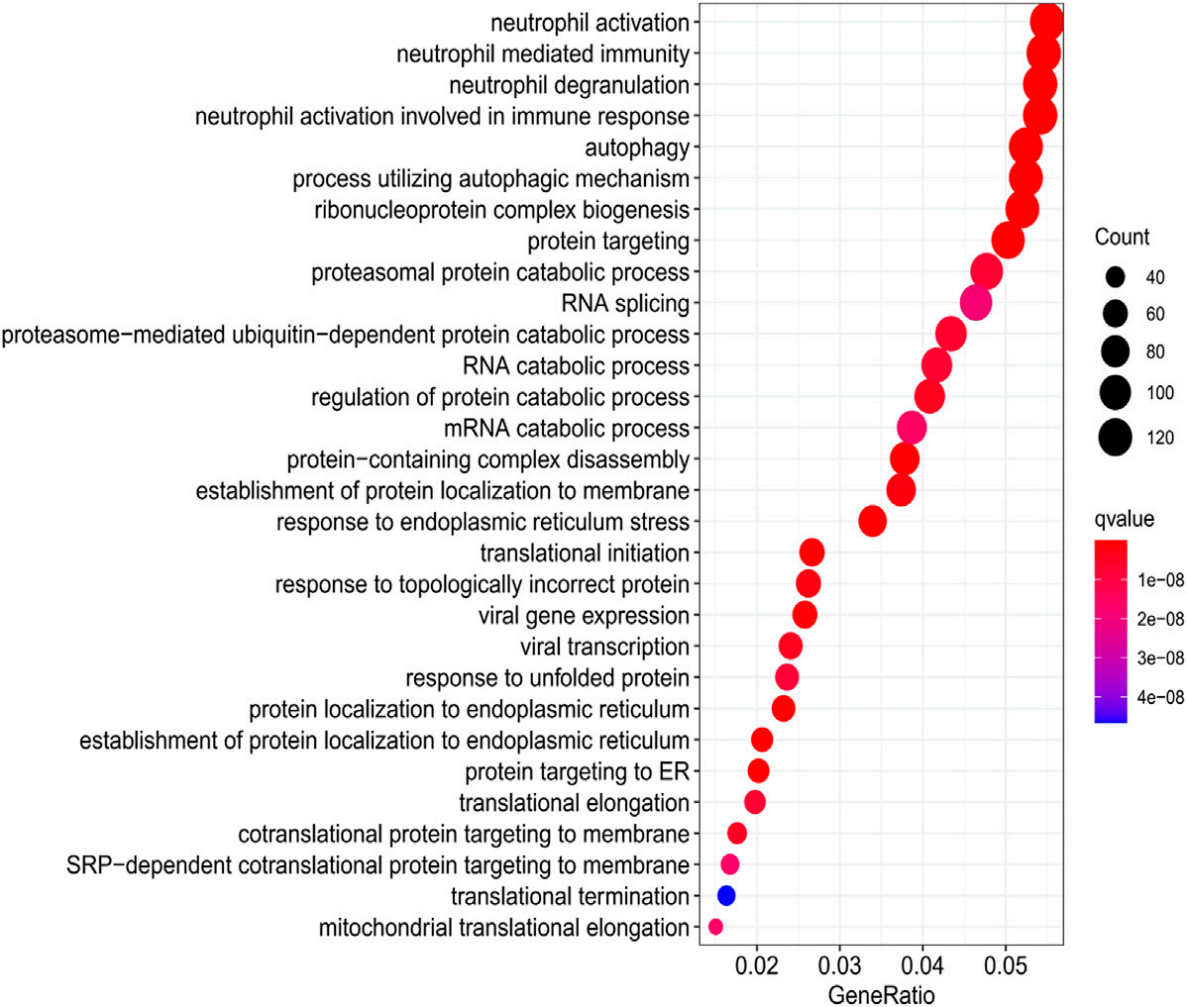
KEGG analysis exhibits the enrichment of *ATP2A1* in colorectal cancer.

Based on the impact of *ATP2A1* on the prognosis of colorectal patients, the regulatory effect of the molecular biology of *ATP2A1* needs to be further explored. Therefore, we applied the GO and KEGG analysis to explore the potential function of *ATP2A1* in colorectal cancer. As Supplementary Figure S4 shows, the most abundant terms in gene ontology (GO) of *ATP2A1* were “neutrophil activation,” “cell-substrate junction,” and “cell adhesion molecule binding,” respectively. Among the KEGG pathways of *ATP2A1*, “neutrophil activation,” “neutrophil-mediated immunity,” “neutrophil degranulation,” and “autophagy” were the most abundant pathways (Figure 5). These results strongly indicated that *ATP2A1* has an intense correlation with tumor immunity, especially neutrophil-mediated immunity.

### Four Small-Molecule Compounds Were Identified Based on the CMap Database

**FIGURE 6:**
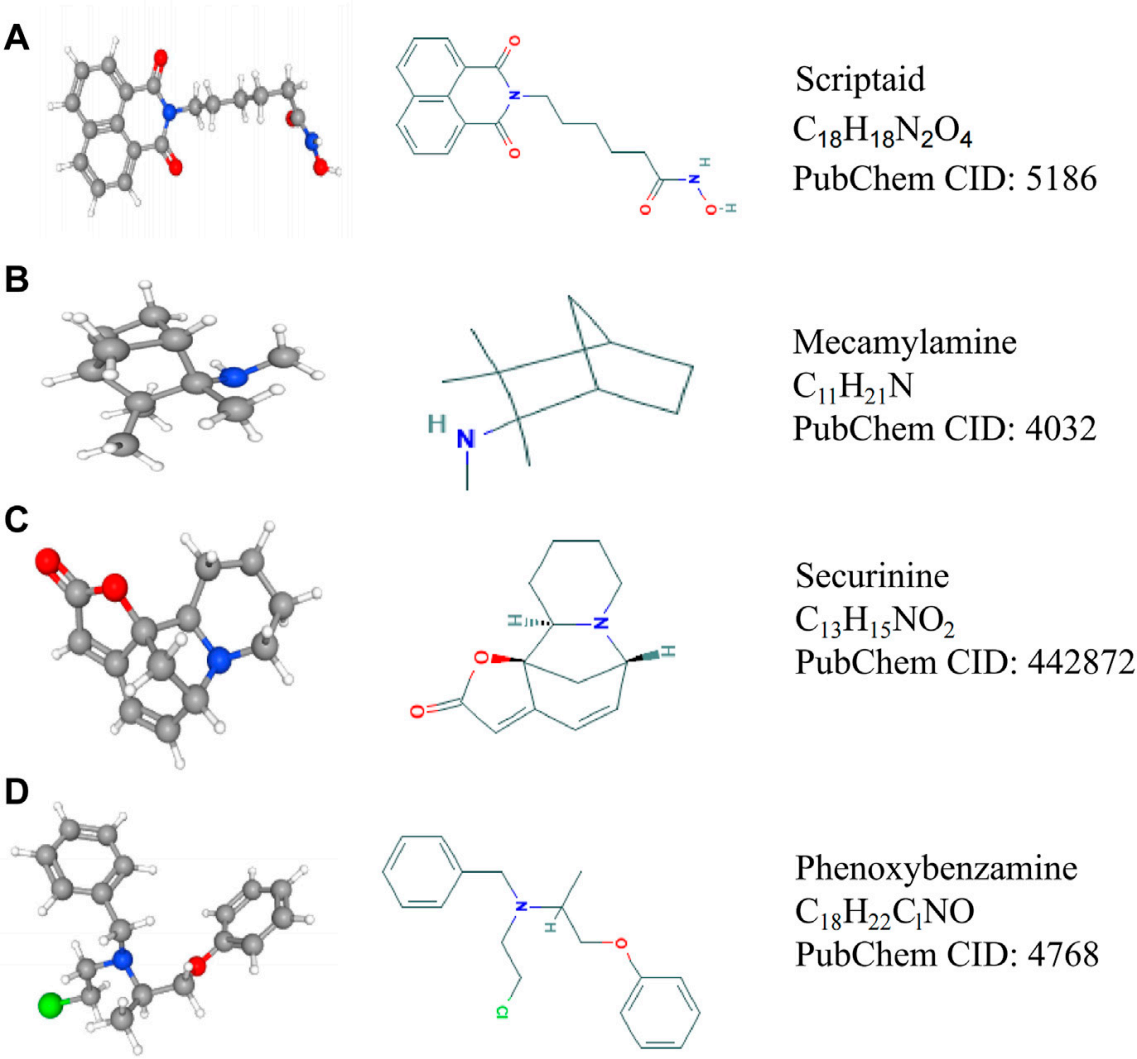
The structure and molecular formula of four small molecule compounds screened by CMap analysis. **(A)** Scriptaid. **(B)** Mecamylamine. **(C)** Securinine. **(D)** Phenoxybenzamine.

The progress of the disease is accompanied by a large number of gene expression disorders, so correcting the gene expression disorder may delay the progress of the disease. The CMap analysis can combine the disorder of the gene expression of the disease with the targeted drugs of genes to correct the disorder; thus, it can organically combine gene expression disorder with disease and drug therapy. Therefore, this study attempts to find drugs that inhibit the expression of *ATP2A1* for reducing the malignancy of CRC cells through the CMap analysis database. Firstly, the genes co-expressed with *ATP2A1* are queried through gene expression analysis and then loaded into the CMap database to obtain the corresponding small molecule drugs. According to screening criteria, four compounds were found to be potential drugs for the inhibition of *ATP2A1* expression. 2D and 3D structure diagrams and molecular formulas are shown in Figure 6.

### To Verify the Effect of Knockdown of *ATP2A1* on the Biological Behavior of Colon Cancer Cells

**FIGURE 7:**
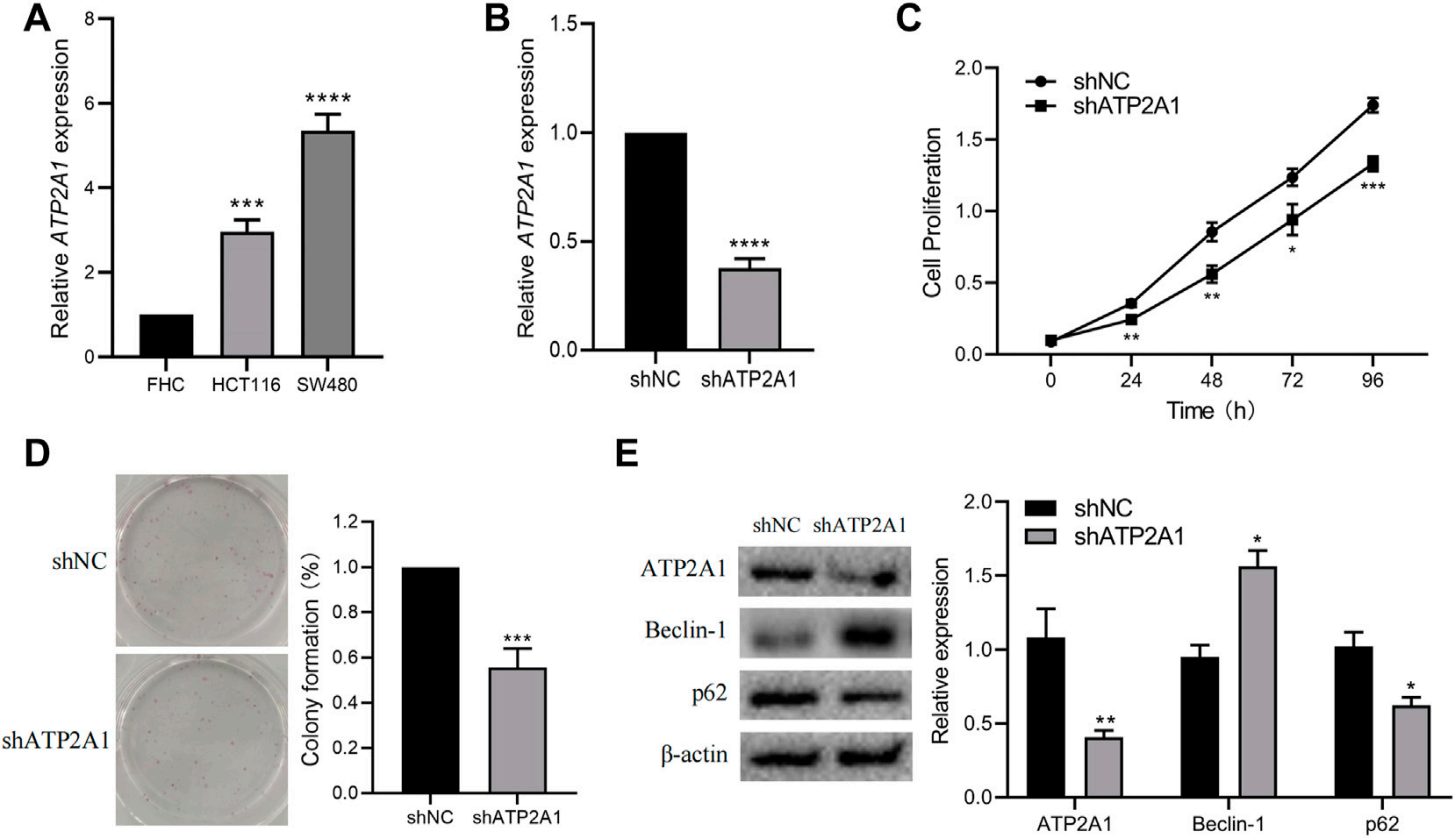
Knockdown of *ATP2A1* inhibits the proliferation of CRC cells and autophagy pathway. **(A)** The mRNA level of *ATP2A1* in FHC, HCT116 and SW480. **(B)** Knockdown of *ATP2A1* in SW480 treated with shRNA. **(C)** The cell proliferation at each time point via CCK8 assay. **(D)** The colonies of SW480 cells with different treatments. **(E)** The expression of key proteins of autophagy pathway in both groups. *p < 0.05, **p < 0.01, ***p < 0.001, ****p < 0.0001 vs. shNC.

First, we used RT-qPCR in two colorectal cancer cells (HCT116, SW480) and colorectal normal mucosal cells (FHC) and found that the expression level of *ATP2A1* was also significantly increased in two colorectal cancer cells, especially in SW480 cells (Figure 7A). Therefore, we selected the SW480 cell for *ATP2A1* knockdown and cell phenotype experiments. Thereafter, we used shRNA to knockdown *ATP2A1* expression by nearly 60% (Figure 7B), and the cell proliferation experiment using CCK8 showed that down-regulation of *ATP2A1* could delay the proliferation rate of cells at 24 h similar at other time points (Figure 7C). Meanwhile, colony formations were also reduced after *ATP2A1* knockdown in contrast to the shNC group (Figure 7D). Lastly, we further explored the mechanism of *ATP2A1* function in colorectal cancer and verified the autophagy pathway by referring to the results of the KEGG analysis. The result showed that after the knockdown of *ATP2A1* expression, the protein level of autophagy-related protein Beclin-1 was significantly up-regulated, and the level of the p62 gene decreased on the contrary (Figure 7E). The above results showed that *ATP2A1* could significantly regulate the autophagy pathway in colorectal cancer, thus affecting the proliferation of colorectal cancer cells.

## Discussion

*ATP2A1* is an important enzyme that maintains intracellular calcium homeostasis. Previous studies mainly focused on its enzyme activity. However, whether *ATP2A1* has other functions has not been reported. In this study, we analyzed the relationship between *ATP2A1* gene expression and the prognosis of colorectal cancer patients using the TCGA database. For the first time, we used sufficient evidence to show that *ATP2A1* was a risk factor for colorectal cancer, and the high expression of *ATP2A1* was associated with the poor prognosis of colorectal cancer patients. Moreover, we also analyzed the correlation between *ATP2A1* methylation level and gene expression and clinical features. We found that the hypermethylation of *ATP2A1* was negatively correlated with tumor progression, which suggested that *ATP2A1* might be an oncogene and plays a role in promoting the progression of colorectal cancer. Additionally, we analyzed the relationship between immune infiltration and *ATP2A1* expression in colorectal cancer patients and found some immune cell populations associated with *ATP2A1* expression. Finally, knockdown of *ATP2A1 in vitro* could significantly reduce the proliferation of colorectal cancer cells. Previous studies have also shown that *ATP2A1* is a pathogenic molecule in tumor tissues. For example, the expression level of *ATP2A1* and *ATP2A3* in breast cancer is significantly higher and closely related to the malignant progression of the tumor, leading to a reduction in the survival time of patients (Christodoulou et al., 2021). SERCA is a protein encoded by *ATP2A1* and plays an important role in regulating intracellular calcium level and the increase of SERCA expression level can promote the malignant progression of cells (Chemaly et al., 2018). The expression of *ATP2A1* in cholangiocarcinomas was significantly increased and correlated with the high immune state in the tumor microenvironment, which can be a high risk factor for the prognosis of patients (Lawal et al., 2021). Collectively, our analysis revealed the prognostic value of *ATP2A1* in colorectal cancer, determined the relationship between *ATP2A1* epigenetic expression and clinical features, and enriched its potential role in tumor immunity of colorectal cancer.

Abnormal DNA methylation is a common phenomenon in cancer, which has been effective targets for diagnosis, treatment, and prognosis (Klutstein et al., 2016; Koch et al., 2018; Pan et al., 2018). In colorectal cancer, methylation characteristics are divided into two parts: global hypomethylation and promoter-specific DNA methylation (Tse et al., 2017). Clinical trials of DNA methyltransferase inhibitors in the treatment of CRCs are in progress. In our study, we investigated the methylation levels of six methylation sites of *ATP2A1* in colorectal cancer. We found that cg08576304 had the lowest methylation level and was negatively correlated with gene expression. Thus, the above can explain the high expression of *ATP2A1* in CRC. In addition, we also found that a high *ATP2A1* methylation level was negatively correlated to the tumor stage. Collectively, all these results show that *ATP2A1* may be an oncogene in CRCs.

Previous studies have shown that tumor-infiltrating immune cells are closely related to tumor progression (Lei et al., 2020). Several immunotherapies are in the preclinical and clinical application (Sabado et al., 2017; van Willigen et al., 2018; Pulendran and Davis, 2020). Tumor-infiltrating immune cells are associated with the clinical features and prognosis of colorectal cancer patients and can be used as biomarkers (Ye et al., 2019). In the present study, we observed that the expression of *ATP2A1* was negatively correlated with immune infiltration of CD8^+^ T cells whether in COAD (*r* = −0.144, *p* = 3.70e–03) or in READ (*r* = −0.249, *p* = 3.12e–03). The same phenomenon occurred in the relationship between copy number variation and immune infiltration. Altogether, these results demonstrate that *ATP2A1* may impact tumor progress partly due to the immune aspect.

The KEGG analysis was used to study the possible signaling pathway of *ATP2A1* in colorectal cancer. The results showed that “neutrophil activation,” “neutrophil-mediated immunity,” “neutrophil degranulation,” “autophagy,” and “proteasomal protein catabolic process” are the most relevant pathways. Increasing evidence shows that inflammation is an important factor in tumor development, and neutrophils play an important role in linking inflammation and tumor, especially in chronic inflammation (Lasry et al., 2016; Mantovani, 2018). The elevated ratio of neutrophils to lymphocytes is considered a risk factor for a poor prognosis of cancer. Furthermore, the differential degranulation of neutrophils may promote the metastasis of tumor cells and provide a new cancer treatment (Mollinedo, 2019). Autophagy is an important way to maintain cell homeostasis. Misfolded proteins, damaged organelles, and other discarded components are encapsulated in autophagosomes and are eventually degraded by lysosomes. Dysregulated autophagy plays a key role in health and disease, especially tumors. Generally, it inhibits tumor initiation and promotes tumor progression. Therefore, targeting autophagy has become a prominent issue in cancer therapy (Levy et al., 2017; Onorati et al., 2018). Ubiquitin proteasome and lysosome systems are the main ways of intracellular protein degradation. Proteasome regulates the physiological process of cells by regulating and degrading intracellular proteins in an extremely stringent way. Abnormalities in the ubiquitin-proteasome system can lead to various diseases, including cancer and neurodegeneration. Thus, targeting the ubiquitin-proteasome system can disclose important therapeutic approaches (Rousseau and Bertolotti, 2018; Narayanan et al., 2020).

Finally, we selected autophagy pathway-related proteins Beclin-1 and p62 for gene function verification of *ATP2A1*. It demonstrated that the expression of Beclin-1 remarkably increased with the down-regulation of *ATP2A1*, and the protein level of p62 was positively related to that of *ATP2A1*. As reported, Beclin-1 and p62 are two promising prognostic markers for CRC (Schmitz et al., 2016). Generally, Beclin-1 expression remains at a low level, but the expression of p62 is the opposite in tumors. When cleaved Beclin-1 increases, it leads to the disruption of the complex consisting of Beclin-1 and p62, which ultimately results in autophagy coordinated apoptosis (Song et al., 2018). Moreover, autophagy-dependent oxidative stress has been reported to affect colitis and tumorigenesis (Liu et al., 2019), indicating that the immune microenvironment regulation and infiltration of the immune cell may be influenced by autophagy. Our results confirm that *ATP2A1* knockdown can regulate synergistic autophagy induction of cell apoptosis, indicating the function of *ATP2A1* in the promotion of CRC. Collectively, our results show that *ATP2A1* may promote colorectal cancer cell proliferation through autophagy.

Our study revealed that *ATP2A1* might be a key pathological factor of colorectal cancer and its potential mechanism of action. For the purpose of reducing *ATP2A1* expression, we performed CMap analysis and found four small-molecule compounds. Scriptaid is a common histone deacetylase inhibitor, which can inhibit the growth of tumor cells and promote their apoptosis (Janaki Ramaiah et al., 2017; Liu et al., 2018; Yao et al., 2018). Securinine can induce cell cycle capture and apoptosis through the mitochondrial pathway in HeLa cells (Stefanowicz-Hajduk et al., 2016). Phenoxybenzamine is an alpha-adrenergic antagonist, which can treat hypertensive crises. In addition, many studies have shown that phenoxybenzamine has the potential to treat cancer (Inchiosa, 2018). Hence, we believe these drugs may be potential chemotherapeutic agents to inhibit the malignant characteristics of CRC cells targeting *ATP2A1*. However, using these drugs in the clinical treatment of colorectal cancer needs more research to confirm and obtain permission for clinical treatment.

There are limitations to this study. First, we could not obtain more regulatory effects in the pathological process of tumors because there are few studies on *ATP2A1* in malignant tumors. However, this is also the innovation of this study, which broadens the molecular mechanisms of the cognitive function of *ATP2A1* in colorectal cancer. Second, as a pathogenic gene, we do not have detailed data to support *ATP2A1* expression changes in the blood of colorectal patients. Finally, this study only partially reveals the regulatory effect of *ATP2A1* in the complex pathological mechanism of colorectal patients, but other regulatory mechanisms of *ATP2A1* in colorectal patients need to be further studied.

## Conclusion

The innovation of this study is to reveal for the first time that the high expression of *ATP2A1* in colorectal cancer can significantly reduce the overall survival time of patients and expand its functional regulation mechanism as a pathogenic molecule. In addition, *ATP2A1* can significantly inhibit the proliferation of colorectal cells by inhibiting the process of autophagy. This study confirmed the relationship between *ATP2A1* and the prognosis of colorectal patients, demonstrated the potential of *ATP2A1* as a key pathological factor that mediates CRC progression and ultimately affects patient prognosis, and identified four small molecule drugs which may inhibit the expression of *ATP2A1*, which might have potential value for delaying the pathological process of colorectal cancer.

## Data Availability

The original contributions presented in the study are included in the article/Supplementary Material, further inquiries can be directed to the corresponding author.
