# Supplementary material for: Increased ATP2A1 Predicts Poor Prognosis in Patients With Colorectal Carcinoma
Source: Front Genet. 2022 Jun 16;13:661348. doi: 10.3389/fgene.2022.661348 (PMC9243465; doi:10.3389/fgene.2022.661348)
Supplement: Supplementary file 1 [file Table1.DOCX]

**Table S1 The detailed clinical features of CRC patients in TCGA database**

| **Covariates** | **Type** | **Total** | **Percentages(%)** |
| --- | --- | --- | --- |
| Age | <=65 | 146 | 43.07% |
|  | >65 | 193 | 56.93% |
| Gender | Female | 159 | 46.9% |
|  | Male | 180 | 53.1% |
| Lymphatic invasion | No | 203 | 59.88% |
|  | Yes | 136 | 40.12% |
| Pathologic_M | M0 | 269 | 79.35% |
|  | M1 | 44 | 12.98% |
|  | M2 | 26 | 7.67% |
| Pathologic_N | N0 | 197 | 58.11% |
|  | N1 | 84 | 24.78% |
|  | N2 | 58 | 17.11% |
| Pathologic_T | T1 | 9 | 2.65% |
|  | T2 | 60 | 17.7% |
|  | T3 | 232 | 68.44% |
|  | T4 | 38 | 11.21% |
| Tumor_stage | stage I | 59 | 17.4% |
|  | stage II | 135 | 39.82% |
|  | stage III | 100 | 29.5% |
|  | stage IV | 45 | 13.28% |
| *ATP2A1* expression | High | 170 | 50.15% |
|  | Low | 169 | 49.85% |
