# Supplementary figures and images for: Increased ATP2A1 Predicts Poor Prognosis in Patients With Colorectal Carcinoma
Source: Front Genet. 2022 Jun 16;13:661348. doi: 10.3389/fgene.2022.661348 (PMC9243465; doi:10.3389/fgene.2022.661348)

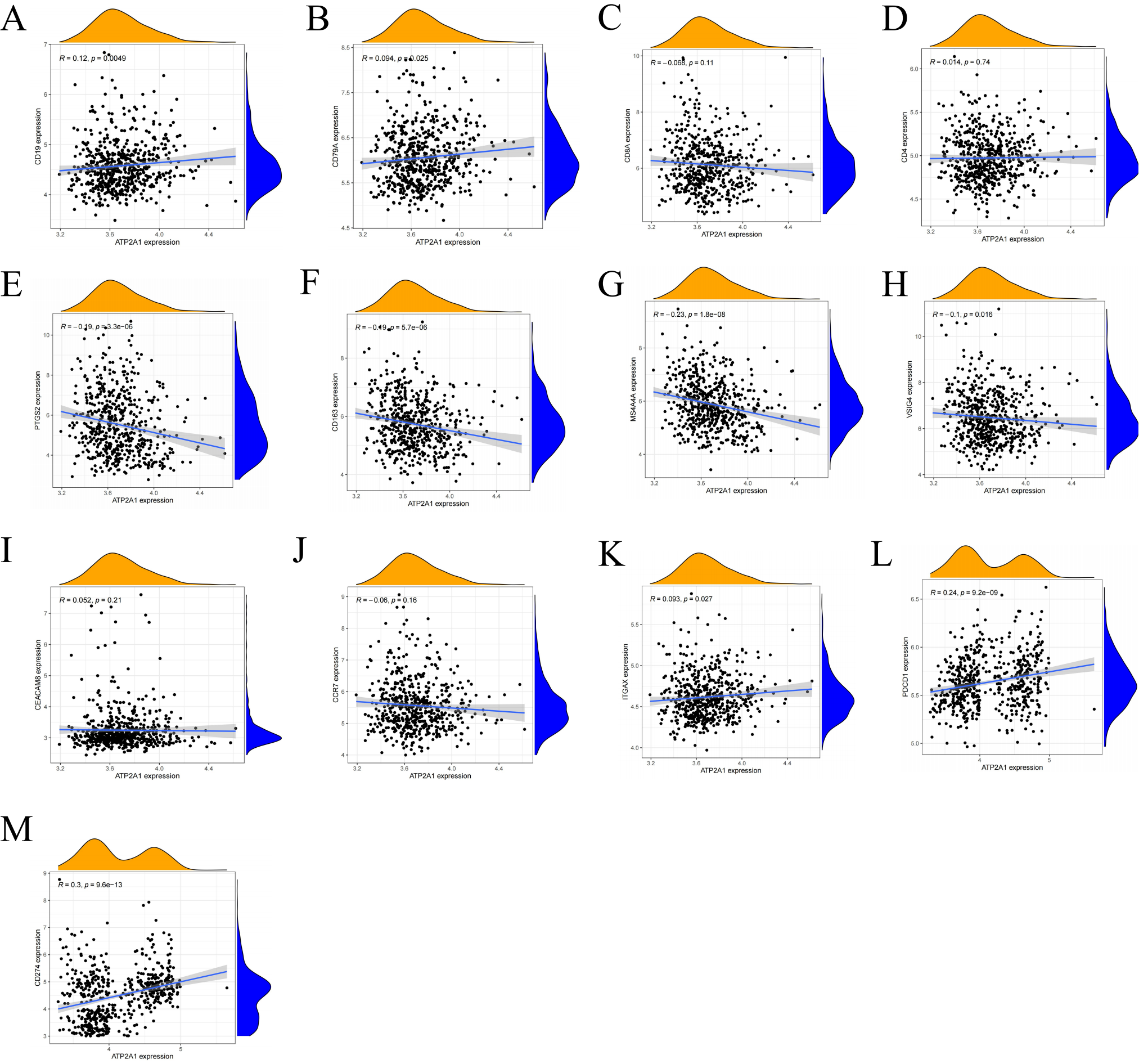

Supplement: Supplementary file 2 [file Image3.TIF]

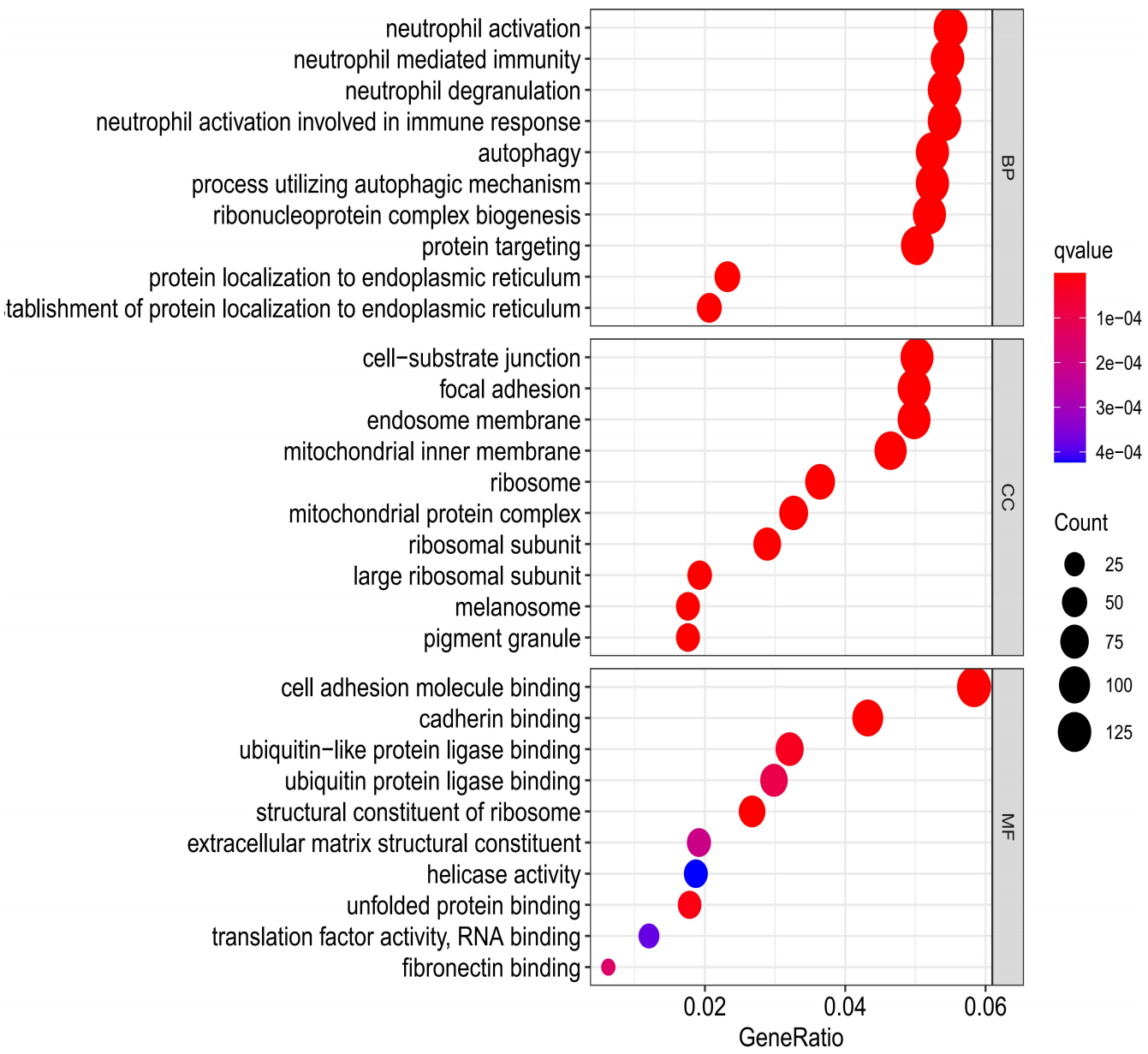

Supplement: Supplementary file 3 [file Image4.TIF]

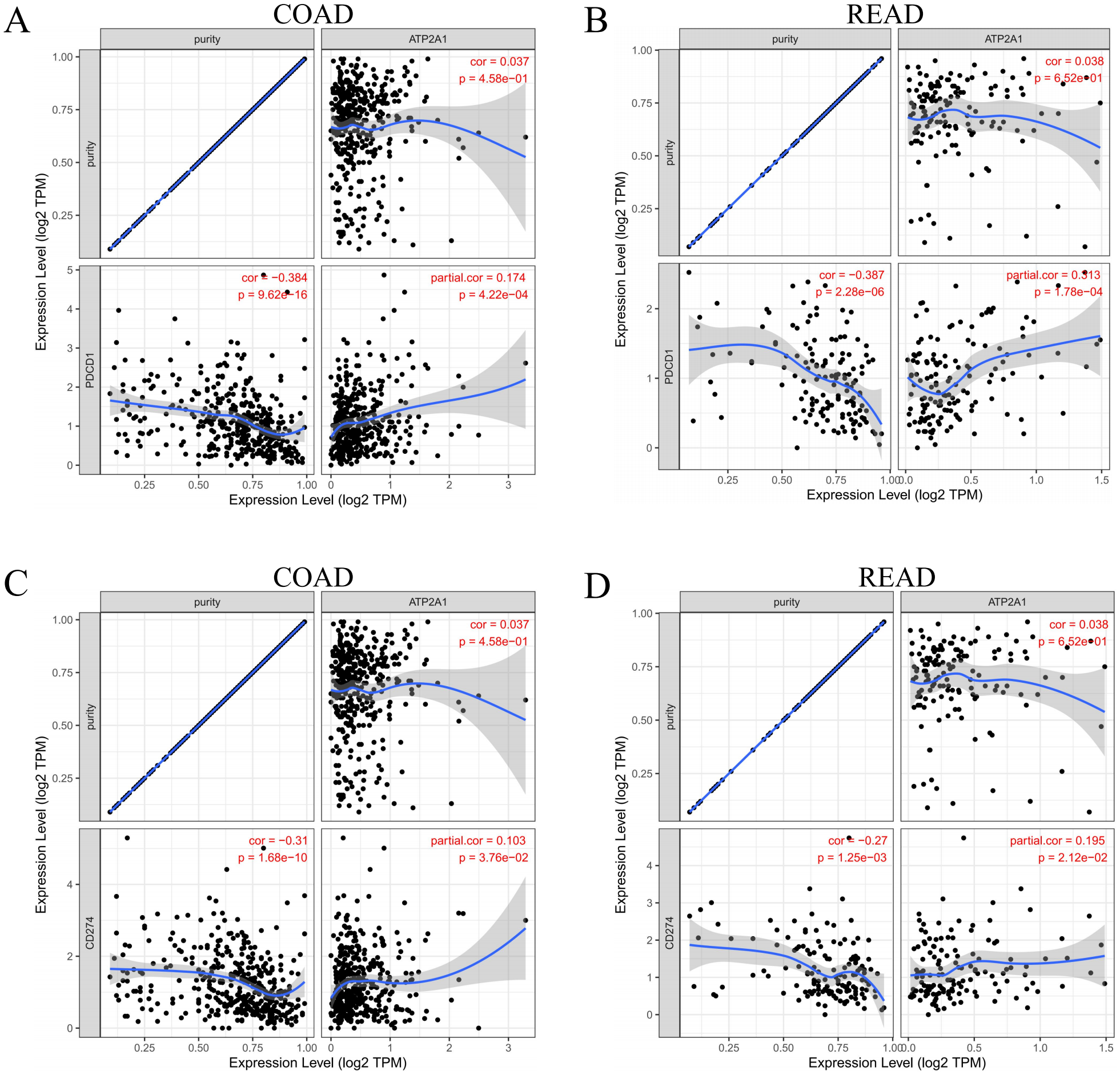

Supplement: Supplementary file 4 [file Image2.TIF]

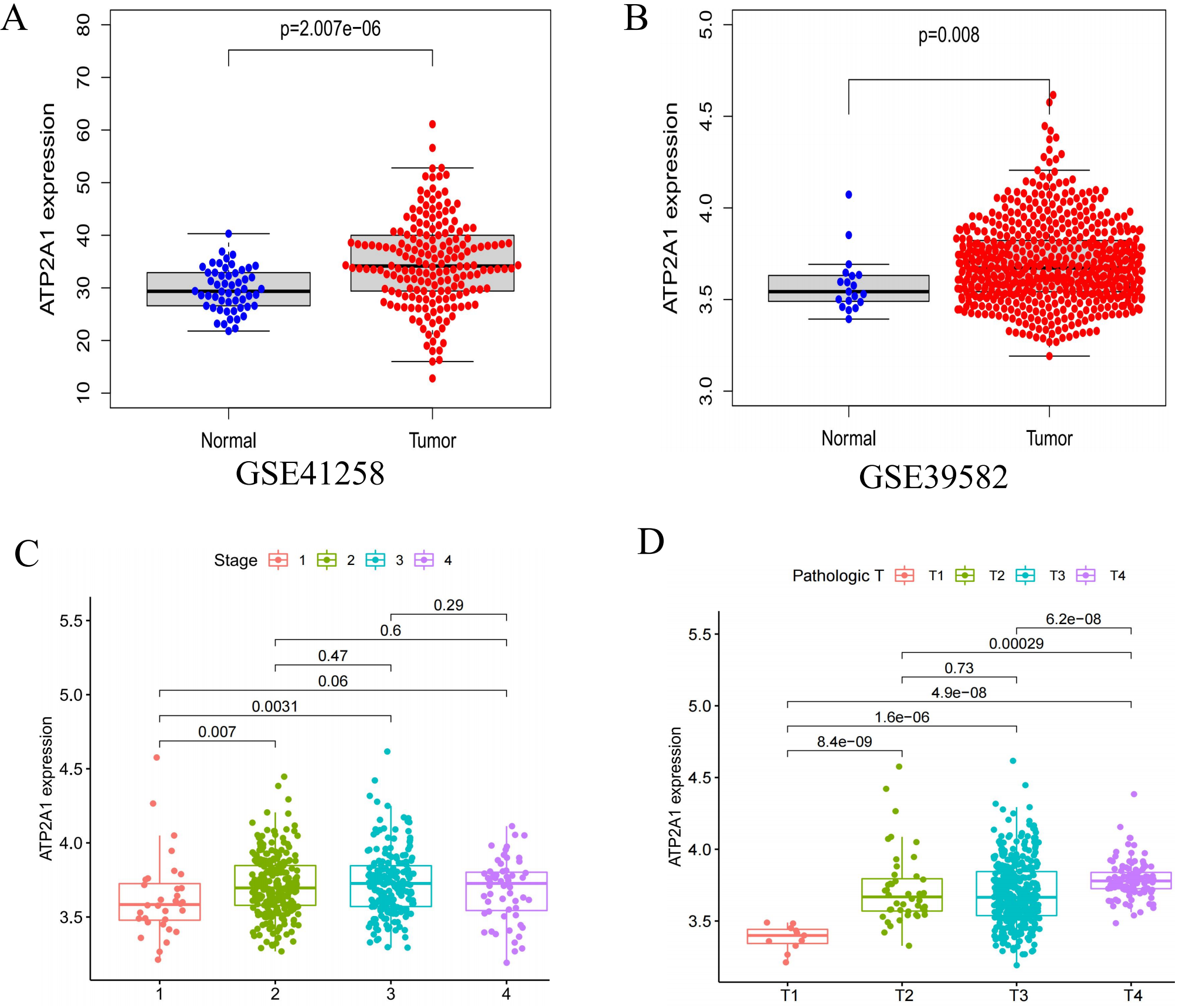

Supplement: Supplementary file 5 [file Image1.TIF]
